# Can BNP-guided therapy improve health-related quality of life, and do responders to BNP-guided heart failure treatment have improved health-related quality of life? Results from the UPSTEP study

**DOI:** 10.1186/s12872-016-0221-7

**Published:** 2016-02-13

**Authors:** Patric Karlström, Peter Johansson, Ulf Dahlström, Kurt Boman, Urban Alehagen

**Affiliations:** Department of Medicine, Division of Cardiology, County Hospital Ryhov, Jönköping, Sweden; Department of Cardiology and Department of Medical and Health Sciences, Linköping University, Linköping, Sweden; Research unit Skellefteå Department of Medicine, Institution of Public Health and Clinical Medicine, Umeå University, Umeå, Sweden

**Keywords:** Heart failure, Treatment guided by natriuretic peptides, Health related quality of life, Responders, BNP, SF-36

## Abstract

**Background:**

To investigate whether B-type natriuretic peptide (NP)-guided treatment of heart failure (HF) patients improved their health related quality of life (Hr-QoL) compared to routine HF treatment, and whether changes in Hr-QoL differed depending on whether the patient was a responder to NP-guided therapy or not.

**Methods:**

A secondary analysis of the UPSTEP-study, a Scandinavian multicentre study using a prospective, randomized, open, blinded evaluation design on patients with HF with New York Heart Association (NYHA) class II-IV. NP-guiding was aimed to reduce BNP <150 ng/L if < 75 years or BNP < 300 ng/L if > 75 years. A responder was defined as a patient with a BNP < 300 ng/L and/or a decrease in BNP of at least 40 % in week 16 compared to study start. Short form-36 (SF-36) was used to measure Hr-QoL. At the study start, 258 patients presented evaluable SF-36 questionnaires, 131 in the BNP group and 127 in the control group. At the study end 100 patients in the NP-guided group and 98 in the control group, presenting data from both the study start and the study end.

**Results:**

There were no significant differences in Hr-QoL between NP-guided HF treatment and control group; however significant improvements could be seen in four of the eight domains in the NP-guided group, whereas in the control group improvements could be seen in six of the domains.

Among the responders improvements could be noted in four domains whereas in the non-responders improvements could be seen in only one domain evaluating within group changes.

**Conclusions:**

Improved Hr-QoL could be demonstrated in several of the domains in both the NP-guided and the control group. In the responder group within group analyses showed more increased Hr-QoL compared to the non-responder group. However, all groups demonstrated increase in Hr-QoL.

## Background

In developed countries, approximately 1-2 % of the adult population has heart failure (HF) and the prevalence is over 10 % among persons older than 70 years of age [1]. There was an increase in the absolute numbers of patients hospitalized because of HF in Sweden between 1990 until 2007, but the age-adjusted prevalence in Sweden has shown a decreasing trend since 2002 [2]. Improved diagnostics, pharmacological therapies and interventions on patients with cardiovascular (CV) diseases have resulted in a decrease in the incidence of HF after acute myocardial infarction in Sweden [3]. However, despite recent improvements in pharmacological treatment, the prognosis is poor, with an estimated five-year survival of approximately 50 % [4]. Moreover, HF patients’ perceived health-related quality of life (HR-QoL) is poorer compared to patients with other chronic diseases [5].

There have been several attempts to improve and individualize HF treatment through guiding with natriuretic peptides (NP). Troughton et al. showed that NP-guided HF therapy reduced all-cause mortality as well as HF and CV hospitalization in patients younger than 75 years [6]. However, only mortality and hospitalization were evaluated, and in a review article by Anker et al. the importance of patient-reported outcomes such as Hr-QoL was emphasized, and it was recommended that these outcomes should be reported in all clinical CV trials, in addition to mortality and morbidity [7]. There are only a limited number of NP-guided studies that have evaluated the impact of NP-guiding on Hr-QoL [8–13]. Bhardwaj et al. showed that NT-proBNP-guided care was associated with greater and more sustained improvement in Hr-QoL compared to standard of care [11]. However, an association between NP-guiding and improvement in Hr-QoL was not reported in five other NP-guiding studies [8–10, 12, 13]. One reason for the differences in study findings may be that not all HF patients seem to benefit from NP-guided therapy, i.e. their NP levels do not decrease despite increased doses of angiotensin-converting enzyme inhibitors, angiotensin II receptor blockers, beta-blockers and mineralocorticoid receptor antagonists. Thus, these patients, defined as non-responders to HF pharmacological treatment, may have obscured the effects of NP-guided therapy on Hr-QoL. Gaggin et al. [14] and the UPSTEP study [15, 16] have previously shown that the responders had significantly reduced risk of CV- and HF hospitalization and CV- and HF mortality compared to non-responders. However, to our knowledge no studies have reported whether responders to NP-guided HF treatment also show beneficial effects of their treatment on Hr-QoL.

### Aim

The primary aim was to investigate whether B-type natriuretic peptide-guided treatment of HF patients compared to routine HF treatment improves the perceived Hr-QoL. The secondary aim was to evaluate whether changes in HR-QoL differed depending on whether the patient was a responder to NP-guided therapy or not.

## Methods

### Population

This study is based on data from the UPSTEP-study and has been described elsewhere [15]. In brief, the UPSTEP study was a Scandinavian multicentre study (15 centres in Sweden and four centres in Norway), on patients with HF with New York Heart Association (NYHA) class II-IV, using a PROBE design (prospective, randomized, open, blinded evaluation) [15]. The patients were randomized into a BNP-guided treatment group (BNP group) and a control group (conventionally treated), (CTR group). In the CTR group, medical treatment was adjusted at the discretion of the investigator based on changes in signs and/or symptoms of worsening HF, in accordance with the guidelines [15].The patients were recruited after an episode of worsening HF and a verified left ventricular ejection fraction (LVEF) < 40 %, NYHA class II–IV and a BNP > 150 ng/L if < 75 years, and >300 ng/L if >75 years, were required to be included. Most patients were in NYHA functional class III (55 %) and 14 % were in NYHA class IV. The mean values of BNP were 808 ng/L in the BNP group and 899 ng/L in the CTR group at the study start (Table 1). Both groups were well treated at baseline regarding HF medication. In the BNP group 146 out of 147 patients (99 %) were receiving treatment with renin-angiotensin system (RAS)-blockers versus 129 of 132 patients (98 %) in the CTR group. Approximately 50 percent of those in both groups had impaired renal function defined as an estimated Glomerular Filtration Rate (eGFR) < 60 mL/min/1.73 m^2^ [15].

**Table 1:** Baseline characteristics for conventionally treated (CTR group) and patients whose treatment was guided by BNP (BNP group) and for non-responders and responders (defined as a patient with a BNP < 300 ng/l and/or a decrease in BNP of at least 40 % in week 16 compared to BNP at study start) (ref art 2)

Characteristics	BNP group	CTR group	p-value	Responders	Non-responders	p-value
	*n* = 147	*n* = 132		*n* = 78	*n* = 53	
Background						
Age years (SD)	71.6 (±9.7)	70.1 (±10)	0.19	69.6 (±10.2)	74.0 (±8.1)	0.009
Gender male, n (%)	107 (73 %)	96 (73 %)	0.99	63 (81 %)	34 (64 %)	0.33
IHD n (%)	81 (55 %)	76 (58 %)	0.68	43 (55 %)	30 (57 %)	0.87
Hypertension n (%)	39 (27 %)	30 (23 %)	0.46	35 (45 %)	25 (47 %)	0.80
Diabetes Mellitus n (%)	39 (27 %)	48 (36 %)	0.08	19 (24 %)	13 (25 %)	0.98
Physical examination						
Heart rate beats/min (SD)	74 (±18)	73 (±19)	0.89	78 (±20)	71 (±15)	0.93
Systolic blood pressure, mm Hg (SD)	126 (±22)	122 (±22)	0.12	126 (±23)	125 (±21)	0.77
Diastolic blood pressure, mm Hg (SD)	75 (±13)	74 (±13)	0.42	75 (±13)	74 (±12)	0.71
NYHA functional classes						
II n (%)	47 (32 %)	36 (27 %)	0.39	28	18	0.09
III n (%)	76 (52 %)	78 (59 %)	0.22	42	27	0.74
IV n (%)	22 (15 %)	18 (14 %)	0.75	8	8	0.41
Medication						
ACEi n (%)	113 (77)	92 (70)	0.17	65 (83)	37 (70)	0.67
ARB n (%)	51 (35)	46 (35)	0.98	23 (29)	23 (43)	0.10
BB n (%)	137 (93)	125 (95)	0.60	75 (96)	48 (91)	0.19
MRA n (%)	81 (55)	78 (59)	0.50	43 (55)	28 (55)	0.80
Echocardiography (LVEF)						
<30 % n	84 (57 %)	76 (58 %)	0.94	50 (64 %)	27 (51 %)	0.13
30-40 % n	63 (43 %)	56 (42 %)	0.94	28 (36 %)	26 (49 %)	0.13
Laboratory results						
BNP ng/l mean (SD)	808 (±676)	899 (±915)	0.34	805 (±704)	778 (±651)	0.82
eGFR mL/min/1.73 m^2^ mean (SD)	61.4 (±20.9)	60.1 (±20.9)	0.59	66.5 (±21.1)	56.1 (±19.2)	0.005
Potassium mmol/l mean (SD)	4.3 (±0.5)	4.2 (±0.5)	0.38	4.3 (±0.50)	4.3 (±0.52)	0.80

Notes: *ACEi* Angiotensin Converting Enzyme inhibitor, *ARB* Angiotensin receptor blockers, *BB* Beta blocker, *BNP* B-type natriuretic peptide, *CTR* Conventionally treated, *eGFR* estimated glomerular filtration rate (MDRD formula), *IHD* Ischemic Heart Disease, *LVEF* Left Ventricular Ejection Fraction, *MRA* mineralocorticoid receptor antagonist, *NYHA* New York Heart Association functional class, *SD* standard deviation

### Definition of a responder

**Fig. 1:**
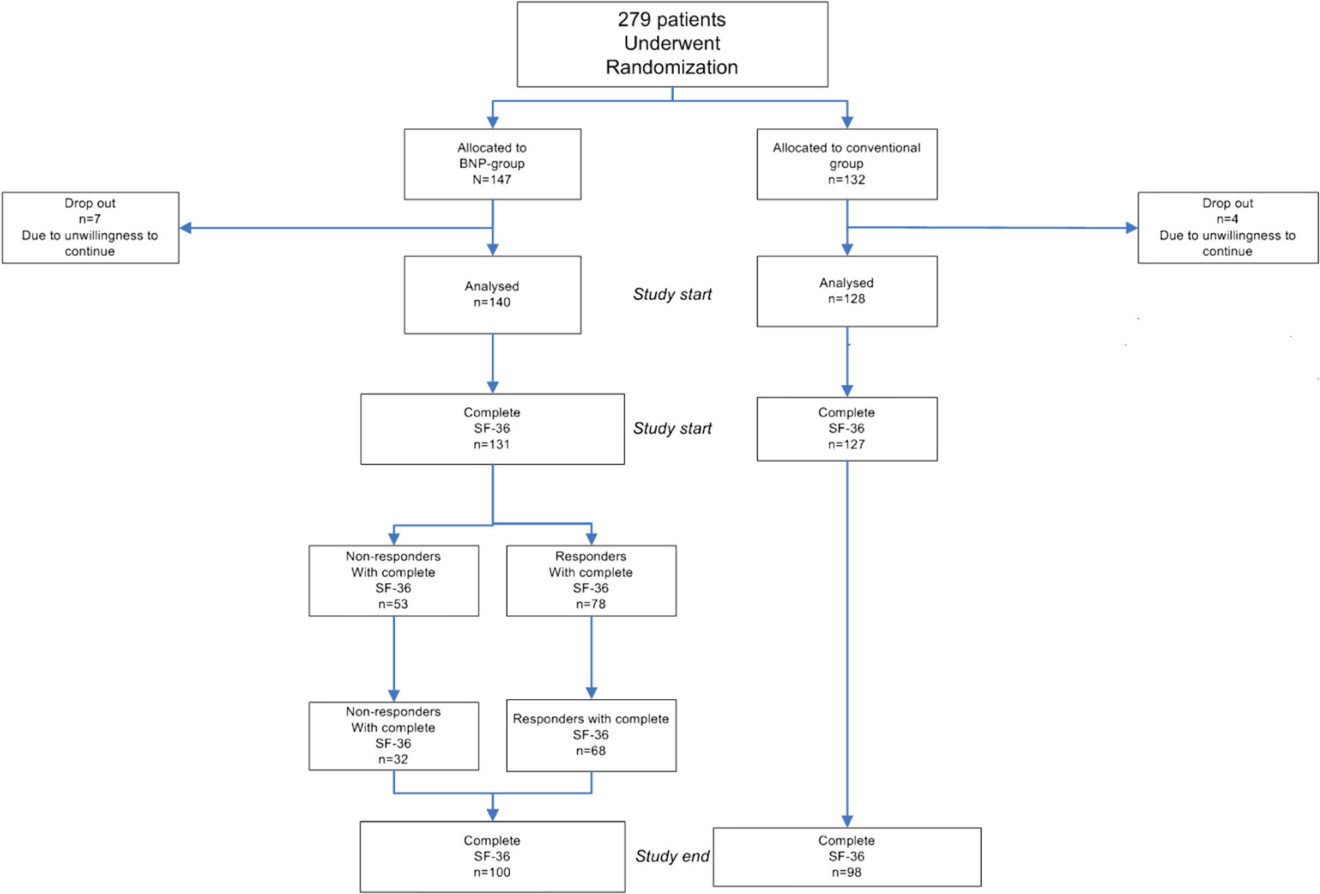
Flow chart of the patients in the UPSTEP-study. Conventionally treated (CTR group) and BNP group, BNP group divided into responders and non-responders. Responder defined as a patient with a BNP under 300 ng/l and/or a decrease in BNP of at least 40 percent in week 16 of follow-up, compared to study start

The definition of a responder was carefully evaluated with different percentage changes in different weeks, and the best definition to a responder was a patient with a decrease in BNP concentration of at least 40 percent in week 16 of follow-up, compared to study start and/or a BNP < 300 ng/L in week 16 [16]. According to that definition, 78 responders (60 %) and 53 non-responders could be identified (Fig. 1). In the conventionally treated group, it was not allowed to control BNP, so responders and non responders are derived from the BNP-group, (Fig. 1). The non-responders had a greater degree of impaired renal function compared to the responders (eGFR 56.1 vs. 66.5; *p* = 0.005) and the non-responders were older (74.0 years vs. 69.6 years; *p* = 0.009) (Table 1). There were no differences in medication, blood pressure, heart rate, serum-potassium or BNP levels between the responders and non-responders at study start [16].

### Health-related Quality of life

The patients’ self-assessed Hr- QoL was measured using the Swedish and Norwegian version of the SF-36 [17, 18]. SF-36 is a well-established Hr-QoL instrument and is frequently used in studies of patients with HF and other diseases [19, 20]. The 36-item instrument includes eight domains of Hr-QoL: physical functioning (PF), role limitations due to physical health problems (RP), bodily pain (BP), general health (GH), vitality (VT), social functioning (SF), role limitations due to emotional health problems (RE) and mental health (MH). The scores were transformed into values between 0-100, with a higher score indicating a better Hr-QoL [21]. The physical component score (PCS) and mental component score (MCS), are two higher order components [17]. All patients filled out the inventory at the study start and at the study end. We used a normative Swedish population evaluated with SF-36 to illustrate the effect ageing has on Hr-QoL. The population 65-74 years consisted of 460 males and 481 females and the population over 75 years of 108 males and 125 females [22].

### BNP analyses

BNP analyses were analysed on site by means of an immunoassay technique (Triage, Biosite Inc., San Diego, CA, USA) as described in a previous publication [15].

## Statistics

Continuous variables were presented as means and standard deviation (SD) values and categorical variables were presented using percentages. Differences in mean values between groups were analysed using the Student’s two-tailed *T*-test for normal distributed data. For non-normal distributed variables, the non-parametric Mann–Whitney U-test was used. Continuous variables were analysed with the student unpaired two-sided *T*-test, whereas the χ ^2^-test was used for discrete variables. Within group differences were analysed with a Wilcoxon paired test. Spearman Rank Order correlations was used to analyse possible correlations between background characteristics and changes in Hr-QoL as indicated by the composite scores PCS and MCS. A p-value <0.05 was considered statistically significant. All calculations were performed on commercial statistical software packages (Statistica v.10,Statsoft Inc, Tulsa, OK, USA).

### Ethical considerations

The study protocol of UPSTEP was approved by the Regional Ethical Committee (Diary nr: M180-04) in Linkoping, Sweden. Every patient signed an informed consent before entering the trial. The study conforms to the Helsinki Declaration.

## Results

### Study sample

The total study population consisted of 279 patients, of whom 147 were randomized to the BNP-guided group and 132 patients to the CTR group. The mean follow-up time of the 198 patients who completed the study was 712 (SD ± 279) days. Eleven patients, seven in the BNP group and four in the control group, discontinued the study for various reasons (Fig. 1), mainly because of unwillingness to continue [15]. At the study start, 258 patients presented evaluable SF-36 questionnaires, 131 in the BNP group and 127 in the control group. As seen in Table 1, no significant differences between the groups regarding baseline characteristics were found. At the study end there were 100 patients in the BNP-guided group and 98 in the control group, presenting evaluable questionnaires from both the study start and the study end.

Of the 147 patients who were randomized to the BNP group in the UPSTEP study, there were 78 responders and 53 non-responders who had answered the Hr-QoL questionnaire at the study start (Fig. 1). The responders had a mean follow-up time of 649 days (SD ± 268) and non-responders 640 (SD ± 365) days (*p* = 0.86). The cohort of patients with SF-36 questionnaires available at both the study start and at the final visit consisted of 68 responders with 675 days (SD ± 266) of follow-up and 32 non-responders with 794 days (SD ± 286) of follow up, *p* = 0.04. The total numbers of worsening HF was 57 in the CTR-group versus 41 in BNP-group, and the amount of serious adverse events was 301 events versus 252 events in BNP group.

### Health-related Quality of life BNP-guided and CTR group

**Fig. 2:**
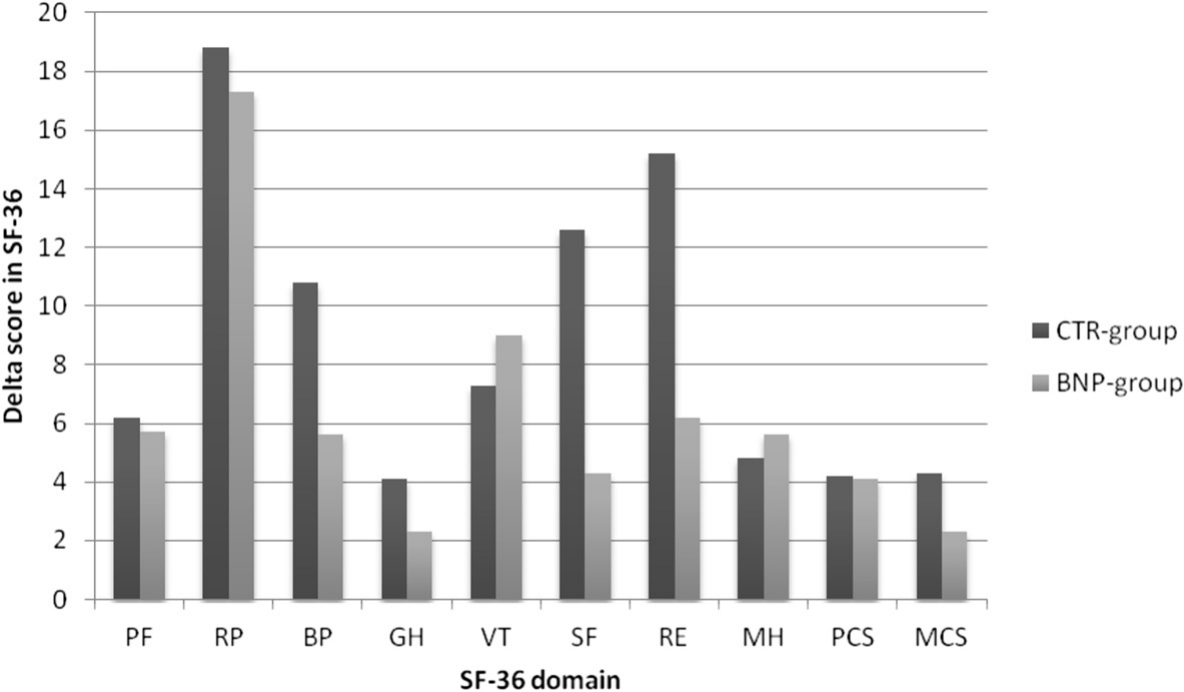
Change in Health-related Quality-of-Life between the NP-guided group (BNP group, *n* = 100) and conventionally treated (CTR group, *n* = 98) group at the study start and the study end regarding different dimensions of the SF-36 questionnaire

Figure 2 presents the change in perceived Hr-QoL during the study. No significant differences between the groups at baseline regarding scores in the eight dimensions could be noted (Appendix 1).

Within-group analyses of changes from the study start showed that significant improvements could be found in four of the eight domains in the NP-guided group (PF, *p* < 0.01, RP, *p* < 0.01, VT, *p* < 0.001 and MH, *p* < 0.01). In the CTR group, significant improvements could be seen in six of the eight domains (RP, *p* < 0.001, BP, *p* < 0.001, VT, *p* = 0.03, SF, *p* < 0.0001, RE, *p* < 0.01, MH, *p* < 0.01) (Appendix 2). For the composite dimension scores PCS and MCS, the NP-guided group improved significantly in PCS, (*p* < 0.001) whereas those with conventional treatment improved in the PCS (*p* < 0.001), and MCS (*p* < 0.002) (Appendix 2).

### Health-related Quality of life responders and non-responders

**Fig. 3:**
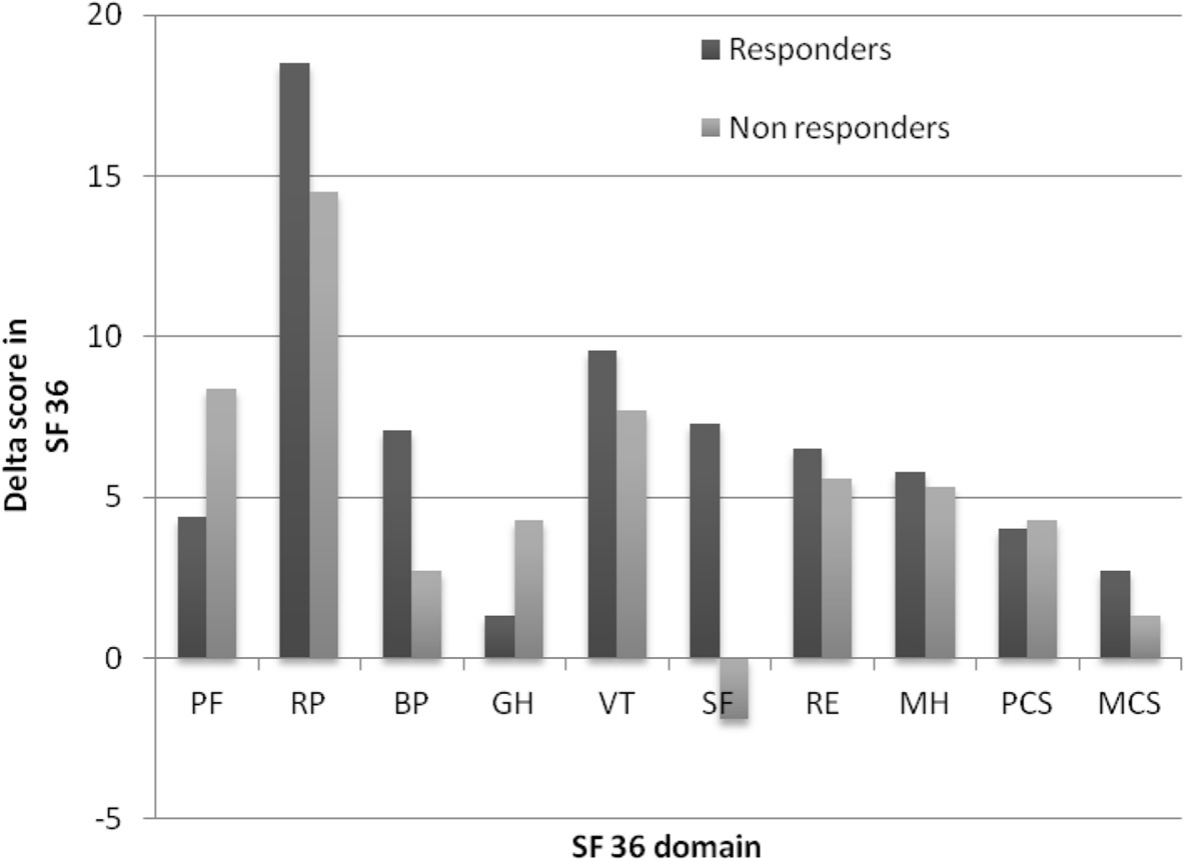
Change in Health-related Quality-of-Life between the responders (*n* = 68) and non-responders (*n* = 32) at the study start and the study end regarding different dimensions of the SF-36 questionnaire

Figure 3 shows the mean changes of the scores between the study start and the study end in the eight different SF-36 domains including the PCS and MCS. Compared to non-responders, the responders at the study start already had significantly better scores in three of the eight SF-36 domains (PF (*p* = 0.001), GH (*p* = 0.01), SF (*p* = 0.006) and in the summary score PCS (*p* = 0.007) (Appendix 1). Within group analyses of changes from the study start showed significant improvements for responders in four domains; RP (*p* < 0.004), VT (*p* < 0.002), SF (*p* = 0.03), MH (*p* = 0.02) and for PCS (*p* < 0.004). For the non-responders the within group analysis was significantly improved in one domain; PF (*p* = 0.03) and for the summary score, PCS (*p* < 0.03) (Appendix 2).

### Correlates of patient characteristics to changes in Health-related Quality of life

Few background characteristics correlated significantly to changes in PCS and MCS. Instead of presenting data on eight dimensions we presented the two summary scores PCS and MCS. In the total study group (i.e. NP-guided and CRT group), a higher heart rate correlated weakly with improvements in PCS (*r* = 0.23) and MCS (*r* = 0.25) whereas a previous myocardial infarct correlated weakly to a worse MCS (*r* = -0.23), In the 100 patients defined as responders or non-responders, no significant correlations were found.

## Discussion

The main finding is that BNP guided HF therapy did not improve the perceived Hr-QoL compared to the conventionally guided HF therapy. However, the BNP group improved in four out of the eight domains, whereas the CTR groups improved in six of the eight domains. We could also demonstrate that the responders improved in four of the eight domains, whereas among the non-responders improvement could only be seen in one single domain. It could also be demonstrated that improvements could be seen irrespective if BNP guided therapy were applied, or if randomized to conventional therapy. Even if a difference could be seen in the size of improvement between responders compared to non-responders, all groups did improve in Hr-QoL. The mechanism behind this could only be speculated on, but it is independent of the use of BNP-guided therapy.

The fact that NP-guided therapy group did not improve in the perceived Hr-QoL compared to the conventionally guided therapy group concurs with studies that have addressed Hr-QoL and NP-guiding [8–10, 12, 13]. However, the Pro-B Type Natriuretic Peptide Outpatient Tailored Chronic Heart Failure (PROTECT) study did present improved HR-QoL [11]. That study showed that those receiving NP-guided medical treatment had a greater and more sustained improvement in HR-QoL compared to those who received conventional handling of their HF. However, our study and the PROTECT study differed in populations and in study design, which may partly explain the differences regarding the perceived Hr-QoL. The patients in the PROTECT study were around 64 years of age, whereas the mean age of our patients was 71 years, thus ours was an older population. As it is documented that with increased age, a decrease in Hr-QoL is to be expected, the margin to influence this variable diminishes as the population is getting older.

We converted our BNP levels to an estimated NT-proBNP level. We found that the UPSTEP population had higher levels of NT-proBNP compared to the population in PROTECT study. Thus, the present study population was older and had higher NT-proBNP concentrations, indicating a more severe HF compared to the PROTECT population. It is therefore possible that our study population was less likely to benefit from the BNP-guided therapy, and thus lesser differences were reported between the two groups in the perceived Hr-QoL. In the evaluation, the score differences between the different groups are small, however as the patients evaluated are elderly and the progress of Hr-QoL can be expected to decrease even small difference can therefore be of importance. In our study we have demonstrated that the responders survive in a higher percentage compared to non-responders [16]. Therefore the demonstration of small differences in domains could be adequate in this patient population.

**Fig. 4:**
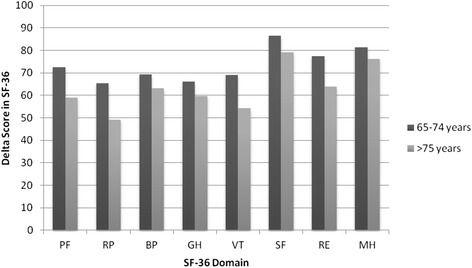
SF 36 scores for a normative Swedish population in the eight domains at age 65-74 years and a population older than 75 years [22], showing decreasing scores in all eight domains with increasing age

It has been discussed in the literature a potential advantage using a disease-specific Hr-QoL instrument in terms of better sensitivity for small changes in HF symptoms compared to a generic instrument. One of the most well-known disease specific instrument in the area of HF is the Minnesota Living with Heart Failure (MLWHF) [11]. However, there are studies indicating is a better ability to differentiate physical and emotional aspects of HR-QoL by SF-36 compared to MLwHF [23]. In the study Trial of Intensified versus standard Medical therapy in Elderly patients with Congestive Heart Failure (TIME-CHF), MLwHF and SF-12, were used to evaluate HR-QoL [9]. The result were consistent with ours, improvement of Hr-QoL within the two groups during the first 12 months, but after that no significant differences between the NP-guided and the control group. This could be interpreted as perceived improvement in Hr-QoL can be detected with both generic and disease specific Hr-QoL instrument if represented by SF-12 and MLWHF. In our study we used the SF-36, but there is no reason to believe that there are fundamental differences in this perspective between the two instruments. Interestingly, all four groups (i.e. the BNP-guided group, the CTR group, responders and non-responders) investigated had improved Hr-QoL. This is interesting since it might be expected that patients with a severe chronic and disabling disease such as HF over time at best may have shown a maintained or a slowly decreased Hr-QoL level. In the Prospective Comparison of ARNI (Angiotensin Receptor–Neprilysin Inhibitor) with ACEI (Angiotensin-Converting–Enzyme Inhibitor) to Determine Impact on Global Mortality and Morbidity in Heart Failure Trial (PARADIGM-HF) study [24], the control group, despite being on optimal treatment, had a significantly decreased Hr-QoL, indicating that Hr-QoL are to be expected to decrease in patient with HF in spite of continuous pharmacologic treatment. In healthy elderly persons the Hr-QoL also decreases as a result of increased age (Fig. 4 ) [22]. In our study, the study population had an age of 71 years at the study start and the follow-up time was almost two years. Instead of an expected decline in the eight domain scores during the study, we saw an improvement in both groups.

HR-QoL is an important tool to determine whether different treatment actions are of any benefit for the patients. Wyrwich et al. evaluated the smallest amount on the SF-36 scale score that would show that a patient moved up or down one response level of the scale items. [25] The smallest points in a state change which represents the smallest amount that an SF-36 scale would change if a patient moved up or down one response level on only one of the SF-36 scale items are; PF 5 points, RH 6.25 points, BP 10 points, GH 5 points, VT 6.25 points, SF 12.5 points, RE 8.33 points and MH 5 points [25]. According to Wyrwich definition the CTR group moved up one response level in PF, RP, BP, VT, SF, RE and the BNP-guided group moved up in PF, RP, VT and MH. The responders moved up in RP, VT, MH and non responders in PF, RP VT and MH. There was no response down in any of the four groups (Table 2).

**Table 2:** The clinically important difference for each SF-36 scale to indicate that the patient moved up (+) or down (-) one response level on only one of the scale items [25] in BNP-guided group, CTR group, responders and non responders

SF-36 scales	Points per state change^a^	CTR group	BNP group	Responders	Non responders
PF	5	+	+	0	+
RP	6.25	+	+	+	+
BP	10	+	0	0	0
GH	5	0	0	0	0
VT	6.25	+	+	+	+
SF	12.5	+	0	0	0
RE	8.33	+	0	0	0
MH	5	0	+	+	+

Abbreviations: *PF* Physical functioning, *RP* Role limitations due to physical health problems, *BP* Bodily pain, *GH* General health, *VT* Vitality, *SF* Social functioning, *RE* Role limitations due to emotional health problems, *MH* Mental health, *BNP* B-type natriuretic peptide, *CTR* conventionally treated group, *SF-36* Short Form 36

Note: ^a^A state change represents the smallest amount that an SF-36 scale score would change if a patient moved up (+) or down (-) one response level on only one of the scales items according to Wyrwich et al.

The exact mechanisms behind the improvements in Hr-QoL are not easy to explain. However, support from health care professionals has been found to be an important factor influencing HF patients’ Hr-QoL [26]. Thus, all patients had scheduled visits at weeks 2, 6, 10, 16, 24, 36, 48 and then every six months until the study end where they met the HF nurse/doctor at the HF clinic. This is more often than clinical routine in the Nordic countries. The mechanism that more use of health resources also influences the perceived Hr-QoL in HF patients has also been reported from primary health care [27, 28]. In the present study NP-guiding has been shown to improve the perceived Hr-QoL in patients with HF. As the perceived Hr-QoL is a complex mechanism other factors also influences the reported result, as seen in this study from the group with conventional HF- treatment where improvement of Hr-QoL was equally reported.

As the design of the present study does not permit the differentiation between effect of the NP-guiding, and the effect of contacts with the HF-professionals, we suggest further research in this important area. From the patient perspective increased Hr-QoL was reported, an important difference compared to what was expected. Therefore more research in this important area is suggested.

### Study limitations

There are limitations to this study that need to be discussed; the relatively small size of the study population restricts the possibilities for drawing conclusions. The population consisted of a homogenous Caucasian population. Therefore it is not possible to draw conclusions about other populations; the age of the study population was high, why the obtained results might be difficult to apply to a general population with HF in the society. The Hr-QoL questionnaire was only filled out at study start and at study end this might influence the possibility to detect changes during the study. The use of a non-disease-specific quality-of-life instrument limits the interpretation of small changes in Hr-QoL.

## Conclusion

Hr-QoL improved in several domains in both the NP-guided therapy group and the control group. Comparing the responder group to the non-responder group, the within group analyses showed improvements in four out of eight domains in the responder group compared to one out of eight domains in the non-responder group.

The perceived Hr-QoL is a complex mechanism that is influenced not only by the pharmacological treatment given, but also by the repeated contacts with health professionals. Both components positively influenced the Hr-QoL in the patients in this study. Therefore more research in perceived Hr-QoL and how to influence it is suggested.

## Appendix 1

**Table 3:** Short Form-36 (SF-36) for conventionally treated (CTR) and patients whose treatment was guided by BNP and for responders and non-responders at study start and at study end. Responder defined as a patient with a BNP under 300 ng/l and/or a decrease in BNP of at least 40 percent in week 16 of follow-up, compared to study start

	Study start			Study end			Study start			Study end		
	Mean (SD)			Mean (SD)			Mean (SD)			Mean (SD)		
	CTR group	BNP group	p	CTR group	BNP group	p	Responders	Non-responders	p	Responders	Non-responders	p
	*n* = 127	*n* = 131		*n* = 98	*n* = 100		*n* = 78	*n* = 53		*n* = 68	*n* = 32	
SF-36												
PF	46.4 (±27)	45.3 (±26)	0.80	54.8 (±26)	55.2 (±27)	0.78	51.3 (±25)	36.5 (±24)	0.001	52.7 (±26)	42.3 (±23)	0.055
RP	19.1 (±33)	28.4 (±38)	0.06	39.8 (±44)	46.4 (±44)	0.21	32.4 (±38)	22.6 (±37)	0.15	33.5 (±44)	22.7 (±38)	0.20
BP	56.4 (±31)	60.6 (±29)	0.26	65.8 (±29)	69.6 (±29)	0.39	62.7 (±29)	57.4 (±29)	0.32	64.6 (±29)	64.2 (±30)	0.95
GH	47.1 (±20)	48.5 (±18)	0.45	50.9 (±20)	53.8 (±19)	0.51	51.8 (±18)	43.8 (±17)	0.01	52.2 (±20)	45.5 (±17)	0.07
VT	43.1 (±22)	42.0 (±21)	0.61	50.2 (±25)	51.9 (±22)	0.63	43.3 (±22)	40.2 (±19)	0.40	42.5 (±25)	43.0 (±18)	0.91
SF	62.4 (±25)	67.8 (±25)	0.10	75.1 (±24)	73.8 (±25)	0.60	72.6 (±25)	60.5 (±23)	0.006	72.6 (±24)	62.5 (±22)	0.056
RE	41.1 (±45)	49.0 (±45)	0.19	54.5 (±44)	57.1 (±42)	0.75	52.8 (±44)	43.4 (±45)	0.24	55.7 (±44)	44.8 (±45)	0.26
MH	70.0 (±20)	69.6 (±19)	0.82	74.1 (±19)	76 (±17)	0.61	70.1 (±18)	68.8 (±20)	0.69	70.1 (±19)	69.5 (±19)	0.87
PCS	31.5 (±11)	32.7 (±11)	0.24	35.6 (±11)	37.8 (±12)	0.18	34.9 (±10)	29.6 (±11)	0.007	35.5 (±11)	31.6 (±11)	0.09
MCS	42.7 (±12)	43.6 (±11)	0.60	46.0 (±11)	46.5 (±10)	0.87	44.3 (±11)	42.7 (±11)	0.45	44.3 (±11)	43.1 (±11)	0.63

Abbreviations: *PF* Physical functioning, *RP* Role limitations due to physical health problems, *BP* Bodily pain, *GH* General health, VT Vitality, *SF* Social functioning, *RE* Role limitations due to emotional health problems, *MH* Mental health, *PCS* Physical component score, *MCS* Mental component score, *SD* Standard Deviation

## Appendix 2

**Table 4:** Changes in Short Form-36 within groups, conventionally treated (CTR) and BNP-guided group and responders versus non-responders. Responder defined as a patient with a BNP under 300 ng/l and/or a decrease in BNP of at least 40 percent in week 16 of follow-up, compared to study start

	Change in Hr-QoL			Within group analysis		Change in Hr-QoL			Within group analysis	
	Mean (SD)					Mean (SD)				
	CTR group	BNP group	p-value	CTR group	BNP group	Responders	Non-responders	p-value	Responders	Non-responders
	*n* = 98	*n* = 100		p-value	p-value	*n* = 68	*n* = 32		p-value	p-value
SF-36										
PF	6.2 (±28)	5.7 (±22)	0.88	0.06	<0.01	4.4 (±22)	8.4 (±22)	0.39	0.06	0.03
RP	18.8 (±46)	17.3 (±49)	0.83	<0.001	<0.01	18.5 (±49)	14.5 (±49)	0.71	<0.004	0.11
BP	10.8 (±32)	5.6 (±34)	0.27	<0.001	0.07	7.1 (±35)	2.7 (±32)	0.55	0.07	0.65
GH	4.1 (±22)	2.3 (±17)	0.52	0.08	0.25	1.3 (±17)	4.3 (±17)	0.40	0.55	0.21
VT	7.3 (±27)	9.0 (±23)	0.64	0.03	<0.001	9.6 (±25)	7.7 (±19)	0.71	<0.002	0.06
SF	12.6 (±28)	4.3 (±27)	0.04	<0.0001	0.13	7.3 (±26)	-1.9 (±29)	0.11	0.03	0.75
RE	15.2 (±46)	6.2 (±47)	0.18	<0.01	0.23	6.5 (±26)	5.6 (±55)	0.93	0.21	0.67
MH	4.8 (±21)	5.6 (±19)	0.77	0.04	<0.01	5.8 (±20)	5.3 (±17)	0.91	0.02	0.12
PCS	4.2 (±11)	4.1 (±9)	0.94	0.001	<0.001	4.0 (±10)	4.3 (±9)	0.87	<0.004	<0.03
MCS	4.3 (±12)	2.3 (±11)	0.25	0.002	0.17	2.7 (±11)	1.3 (±12)	0.57	0.10	0.91

Abbreviations: *PF* Physical functioning, *RP* Role limitations due to physical health problems, *BP* Bodily pain, *GH* General health, *VT* Vitality, *SF* Social functioning, *RE* Role limitations due to emotional health problems, *MH* Mental health, *PCS* Physical component score, *MCS* Mental component score, *SD* Standard Deviation

## Abbreviations

ACEI: Angiotensin-Converting–Enzyme Inhibitor
ARNI: Angiotensin Receptor–Neprilysin Inhibitor
BNP: B-type natriuretic peptide
BNP-group: BNP-guided group
BP: bodily pain
CTR-group: conventional treated group
CV: cardiovascular
GH: general health
HF: heart failure
Hr-QoL: health related quality of life
MCS: mental component score
MH: mental health
MLWHF: Minnesota Living with Heart Failure
NP: natriuretic peptide
NYHA: New York Heart Association
PARADIGM-HF: prospective comparison of ARNI with ACEI to determine impact on global mortality and morbidity in heart failure
PCS: physical component score
PF: physical functioning
PROBE: prospective, randomized, open, and blinded evaluation
PROTECT: Pro-B Type Natriuretic Peptide Outpatient Tailored Chronic Heart Failure
RE: role limitations due to emotional health problems
RP: role limitations due to physical health problems
SD: standard deviation
SF: social functioning
SF-36: Short Form 36
TIME-CHF: The Trial of Intensified vs Standard Medical Therapy in Elderly Patients With Congestive Heart Failure
UPSTEP: Use of PeptideS in Tailoring hEart failure Project
VT: vitality
